# PLOD3 Is Associated with Immune Cell Infiltration and Genomic Instability in Colon Adenocarcinoma

**DOI:** 10.1155/2021/4714526

**Published:** 2021-06-18

**Authors:** Xianyu Deng, Yun Pan, Muqing Yang, Ying Liu, Jiyu Li

**Affiliations:** ^1^Colorectal Cancer Center, Tenth People's Hospital of Tongji University, Tongji University School of Medicine, China; ^2^Center for Difficult and Complicated Abdominal Surgery, Tenth People's Hospital of Tongji University, Tongji University School of Medicine, China; ^3^Department of General Surgery, Tenth People's Hospital of Tongji University, Tongji University School of Medicine, China

## Abstract

Procollagen-lysine, 2-oxoglutarate 5-dioxygenases (PLODs) are a family of enzymes. However, the clinical and functional roles of PLOD3 in colon adenocarcinoma (COAD) have not been investigated. The present study found that PLOD3 was highly upregulated in COAD, which may be resulted from its aberrant DNA methylation. The upregulation of both PLOD3 mRNA and protein was confirmed in our tissue samples. Moreover, high PLOD3 was identified to be associated with unfavorable prognosis in COAD. As genome instability is a hallmark of cancer, PLOD3 was expressed higher in COAD samples with high chromosomal instability (CIN-high) than those with low CIN (CIN-low) and higher in those with low MSI than high MSI, indicating that PLOD3 expression was associated with tumor genomic instability. Furthermore, immune cells showed significantly different infiltrating levels between the high and low PLOD3 expression groups, and the immune score was negatively correlated with PLOD3 expression and higher in samples with low PLOD3 expression, suggesting that high PLOD3 expression was associated with reduced immune cell infiltrating levels in COAD. To further uncover the underlying mechanism of PLOD3 in PLOD3, we compared the COAD samples of high PLOD3 expression with those of low PLOD3 expression and found that high expression of PLOD3 was associated with reduced expression of immune regulators and enhanced activities of two tumor-promoting pathways, including gluconeogenesis and TGF-beta signaling in epithelial-mesenchymal transition (EMT), suggesting that high expression of PLOD3 causes poor prognosis in COAD by weakening the immune cell infiltration and enhancing activities of tumor-promoting pathways. In summary, the present study highlights the importance of PLOD3 and provides the evidence about the functional role of PLOD3 in COAD.

## 1. Introduction

Colon cancer is one of the most prevalent malignancies worldwide. It is estimated globally that colorectal cancer would account for 6.1% of new cancer cases and for 9.2% of cancer-related deaths [1]. Heritable factors are not responsible for most colorectal cancer cases, and chronic intestinal inflammation has been widely detected in colon cancer patients, while environmental mutagens and modifiable factors like unhealthy diet and lifestyle are related to the occurrence of colorectal cancer [2, 3].

A previous study has demonstrated a systemic immune-inflammation index for colorectal cancer using the total number of neutrophils, platelets, and lymphocytes, which could serve as a prognostic indicator for predicting overall survival and help identify patients of higher risk [4]. Of note, glycoproteins are critical players in the cellular and humoral immune system, participating in the assembly of major histocompatibility complex (MHC) antigens, T cell recognition of antigen-presenting cells, and the classical antibody-antigen recognition by immunoglobulins [5]. Meanwhile, glycosylation, as an important posttranslational modification, is associated with multiple fundamental mechanisms in cancer development and progression other than immune modulation, further suggesting that glycoproteins and related genes could be potential biomarkers and drug targets in cancer diagnosis and management [6].

One of those glycoproteins is procollagen-lysine, 2-oxoglutarate 5-dioxygenase 3, which is encoded by PLOD3, and it is an enzyme essentially involved in the biosynthesis of several collagens and glycosylation activity [7]. Emerging evidence suggests that PLOD3 is associated with tumorigenesis in various cancer types. PLOD3 knockdown could inhibit tumor growth in lung cancer through regulating the PKC-delta signaling pathway [8], and also in lung cancer, PLOD3 is found to interact with STAT3 immunosuppressive signals, which promotes lung cancer metastasis via dysregulated RAS-MAP kinase pathway [9]. Moreover, it is reported that PLOD3 downregulation would lead to decreased expression levels of TWIST1, further resulting in the inhibition of *β*-catenin and AKT signaling and suppressing the progression of renal cell carcinoma [7]. In addition, a recent study has implied that MYC and TWIST1 are essential for the activation of innate immunity and cellular invasion, as the recruitment and polarization of tumor-associated macrophages (TAMs) require a certain cytokinome elicited by them, and overexpression of TWIST1 could promote metastasis in hepatocellular carcinoma [10]; meanwhile in another study, inactivation of PLOD3 is found to inhibit in vitro and in vivo liver tumorigenesis in hepatocellular carcinoma [11]. Such findings hint an underlying association between PLOD3 and antitumor immunity, and such association may as well exist in the context of colon cancer, which we hope to elucidate in this study.

## 2. Materials and Methods

### 2.1. Public Datasets

The count-based mRNA expression data and protein expression data were obtained from UCSC Xena (http://xena.ucsc.edu/) and LinkedOmics (http://www.linkedomics.org/), respectively. The mRNA expression data was prenormalized by fragment per kilo-million reads (FPKM), and the protein expression was quantile-normalized for further analysis.

### 2.2. Clinical Sample Preparation

Six pairs of fresh colon adenocarcinoma and adjacent normal tissues were collected from Tenth People's Hospital of Tongji University, Tongji University School of Medicine, which was approved by the Human Research Ethics Committee of this hospital. The written informed consent was collected from each patient. All samples were stored in −80°C for the following experiments.

### 2.3. Quantitative Real-Time Polymerase Chain Reaction (qPCR)

The tissue samples were lysed using a Trizol reagent (Invitrogen, USA). Following the manufacturer's protocol, we performed the reverse transcription using RevertAid First Strand cDNA Synthesis Kit (Thermo, Fermentas, USA). The mRNA expression was quantitatively analyzed using an ABI Stepone plus (StepOnePlus™). GAPDH is an internal reference. The primers for PLOD3 are as follows: forward: 5′- CTGAAGAAGTTCGTCCAGAGTG-3′ and reverse: 5′- ACCGATGAATCCACCAGAATTG-3′; the primers for GAPDH are as follows: forward: 5′- GGAGCGAGATCCCTCCAAAAT-3′ and reverse: 5′- GGCTGTTGTCATACTTCTCATGG-3′. All these experiments were conducted in triplicates. The average values were used as the RNA expression levels of the samples.

### 2.4. Western Blot

Total protein was isolated from tissue samples using RIPA lysis buffer (Beyotime Biotechnology, China) and then quantified by the BCA assay kit (Beyotime Biotechnology, China). Each 20 *μ*g total protein was electrophoresed on 10% SDS-polyacrylamide gel and then transferred to polyvinylidene fluoride (PVDF) membranes (Millipore, USA). The primary antibodies used were anti-PLOD3 (Proteintech, China) and anti-GAPDH (Cell Signaling Technology, USA). Membranes were then washed three times in TBST solution for 15 min each and then incubated with secondary antibodies for 1 h.

### 2.5. Two-Sample and Multisample Comparisons and Survival Analysis

The two-sample and multisample comparisons were conducted using the Wilcoxon rank sum test and Kruskal-Wallis test, respectively. The tumor samples were stratified into high and low groups using the median expression of PLOD3 as the threshold. The Cox proportional hazard regression model was built to fit the survival time/status with the PLOD3 expression, and the log-rank test was used to evaluate the statistical significance.

### 2.6. Estimation of Immune Cell Proportion

The immune cell proportions were estimated using TIP (tracking tumor immunophenotype) [12], which used CIBERSORT [13] and marker genes of 14 immune cell types. The mRNA expression was normalized to transcript per million reads (TPM) using R scater package and coding length of gene. The TPM-based mRNA expression was used as the input in TIP. The immune scores were estimated by ESTIMATE (Estimation of STromal and Immune cells in MAlignant Tumours using Expression data) [14].

### 2.7. Gene Set Enrichment Analysis

The genes were ranked by the correlation coefficients between PLOD3 and the remaining genes using TCGA mRNA expression data. The enrichment scores for genes of immune regulators and those involved in signaling pathways were calculated based on the ranks and correlation coefficients of these genes. A total of 1000 times permutation was used to evaluate the statistical significance of the enrichment score and the immune regulators and pathways. This analysis was implemented in R clusterProfiler package [15].

## 3. Results

### 3.1. The RNA and Protein Expression of PLOD3 Are Upregulated in COAD

**Figure 1:**
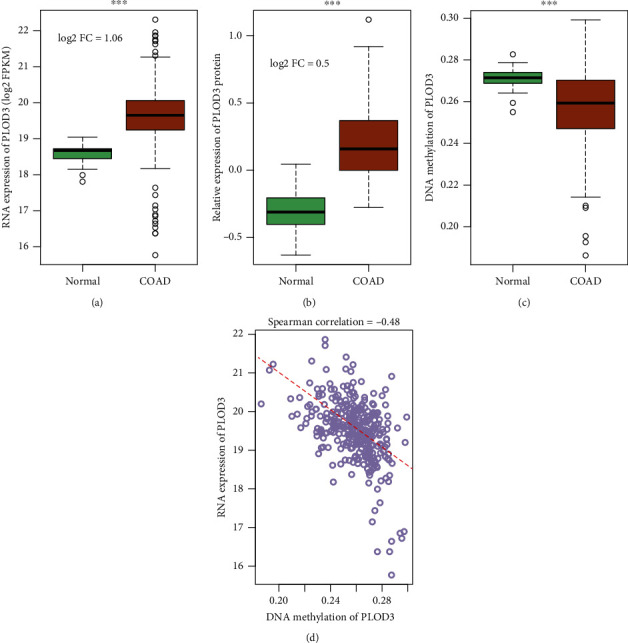
PLOD3 is upregulated in COAD. The RNA and protein expression of PLOD3 are upregulated in the COAD samples of (a) TCGA and (b) CPTAC cohorts, respectively. (TCGA_(tumor)_ = 469, TCGA_(normal)_ = 41, CPTAC_(tumor)_ = 97, and CPTAC_(normal)_ = 100). (c) The PLOD3 promoter is hypomethylated in COAD samples of TCGA cohort. (d) The promoter DNA methylation and RNA expression of PLOD3 are negatively correlated in TCGA cohort. The line was fitted by using RNA expression and DNA methylation of PLOD3 as response and predictor variables, respectively. The symbol ^∗∗∗^ indicates the *P* value <0.001 (log2 FC: log2 fold change).

To investigate the expression pattern of PLOD3 in colon adenocarcinoma (COAD), we collected mRNA expression data from The Cancer Genome Atlas (TCGA) [16] and protein expression data from Clinical Proteomic Tumor Analysis Consortium (CPTAC) [17]. Specifically, PLOD3 mRNA was found to be highly upregulated in COAD of TCGA cohort as compared with the adjacent normal tissues (Figure 1(a), *P* value <0.001). Consistently, the PLOD3 protein expression was also upregulated in COAD tissues of CPTAC cohort (Figure 1(b), *P* value <0.001). Moreover, we also collected the DNA methylation data of TCGA cohort and observed that the promoter DNA methylation levels of PLOD3 were decreased in COAD samples (Figure 1(c), *P* value <0.001). The correlation analysis revealed that the promoter DNA methylation of PLOD3 was negatively correlated with its mRNA expression (Figure 1(d), Spearman correlation = −0.48), suggesting that the dysregulation of PLOD3 was associated with its promoter hypomethylation.

### 3.2. Validating the Upregulation of PLOD3 in COAD and Adjacent Normal Tissues

**Figure 2:**
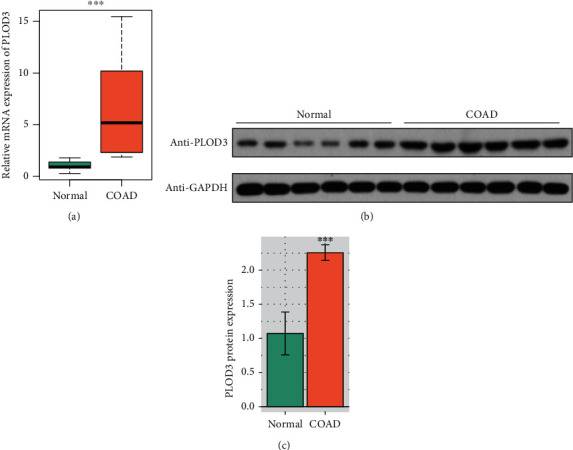
Validation of PLOD3 upregulation in COAD and adjacent normal tissue samples. (a) The mRNA expression of PLOD3 is upregulated in the COAD tissue of our cohort using the qPCR method (*n* = 6). (b, c) The protein expression of PLOD3 is upregulated in COAD samples of our cohort using Western blot. The symbol ^∗∗∗^ indicates the *P* value <0.001.

To validate the upregulation of PLOD3 in COAD and adjacent normal tissues, we collected six pairs of COAD and normal tissues and detected the mRNA and protein expression levels in these samples. The mRNA expression was also upregulated in the COAD samples compared with the adjacent normal tissues using the qPCR method (Figure 2(a), *P* value <0.001). Consistently, the Western blot assay confirmed the upregulation of PLOD3 protein in COAD (Figures 2(b) and 2(c), *P* value <0.001). These results further demonstrated high expression of PLOD3 in COAD.

### 3.3. High Expression of PLOD3 Indicates Poor Prognosis in COAD

**Figure 3:**
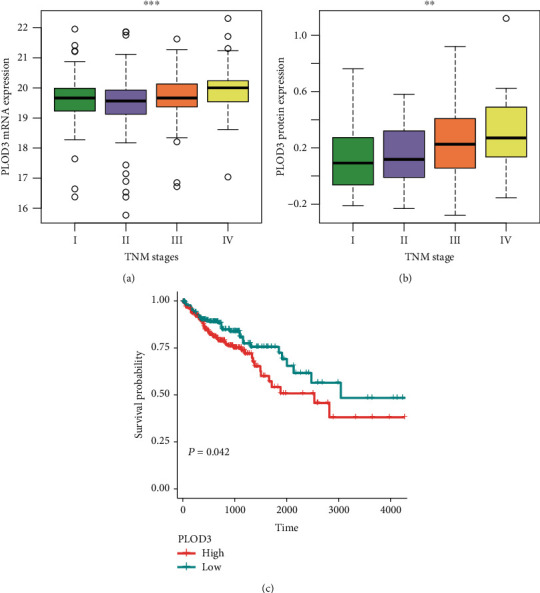
PLOD3 expression is increased with the progression of COAD. The (a) RNA and (b) protein expression are increased in advanced stages of COAD (TCGA_(stage I)_ = 78, TCGA_(stage II)_ = 182, TCGA_(stage III)_ = 131, TCGA_(stage IV)_ = 65, CPTAC_(stage I)_ = 10, CPTAC_(stage II)_ = 39, CPTAC_(stage III)_ = 40, and CPTAC_(stage IV)_ = 8). (c) COAD patients with high expression of PLOD3 have shorter overall survival than those with low expression of PLOD3. The symbols of ^∗^, ^∗∗^, and ^∗∗∗^ indicate the *P* values <0.05, 0.01, and 0.001, respectively.

As PLOD3 was highly upregulated in COAD samples, we then investigated whether its expression was associated with COAD prognosis. The comparison of the tumor samples with distinct TNM stages revealed that PLOD3 was increased in the advanced TNM stages in both TCGA and CPTAC cohorts (Figures 3(a) and 3(b), *P* value <0.01). Furthermore, we stratified the tumor samples into two groups by the PLOD3 expression (high vs. low). The comparison of the two tumor groups indicated that the high group had significantly shorter overall survival time than those of low group (Figure 3(c), *P* value <0.05). These results indicated that PLOD3 expression was associated with poor prognosis in COAD.

### 3.4. PLOD3 Expression Is Associated with Tumor Genomic Instability

**Figure 4:**
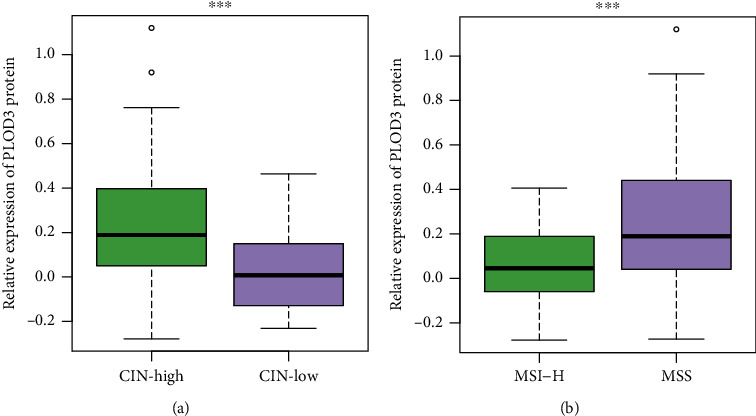
PLOD3 is associated with genomic instability. PLOD3 is expressed higher in COAD samples with (a) high chromosomal instability (CIN-high) or (b) low microsatellite instability (MSI-low) (CIN-high vs. CIN-low: 75 vs. 20 and MSI-high vs. MSI-low: 22 vs. 73). The symbol ^∗∗∗^ indicates the *P* value <0.001.

The genomic instability in cancer includes abnormal chromosomal instability and microsatellite instability (MSI), which are characterized by widespread imbalances in chromosome number (aneuploidy) or loss of heterozygosity [18] and hypermutable phenotype caused by the loss of DNA mismatch repair activity [19]. To further investigate the association between PLOD3 and some genomic features, we found that PLOD3 was expressed higher in COAD samples with high chromosomal instability (CIN-high) than those with low CIN (CIN-low) (Figure 4(a), *P* value <0.001). In contrast, PLOD3 was expressed lower in COAD samples with high microsatellite instability (MSI-high) than those with low MSI (MSI-low) (Figure 4(b), *P* value <0.001). These results indicated that PLOD3 expression was associated with tumor genome instability.

### 3.5. PLOD3 Expression Is Associated with the Immune Cell Infiltration

**Figure 5:**
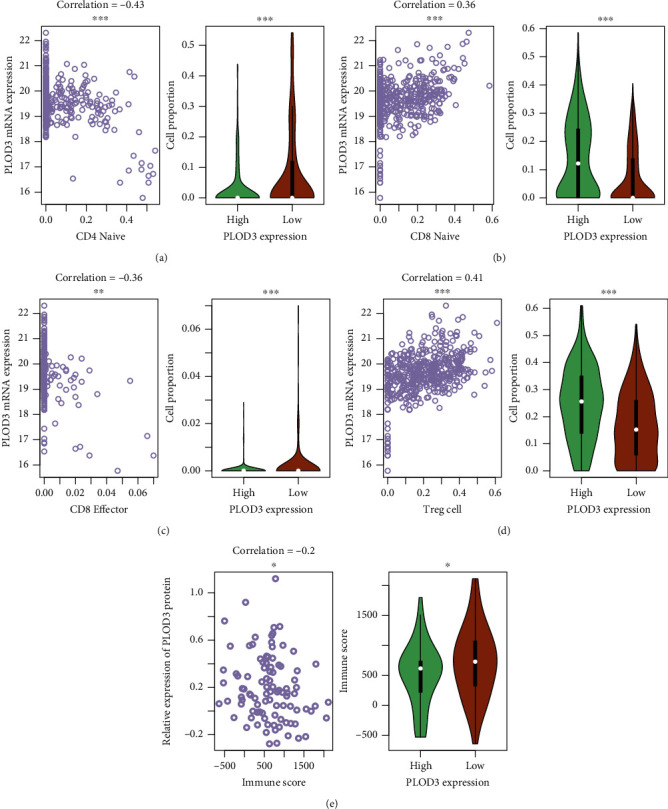
Correlation between immune cell infiltration and PLOD3 expression. The correlation between PLOD3 expression and the proportions of tumor-infiltrating immune cells, including CD4 naïve, CD8 naïve, CD8 effector, and Tregs, and immune scores are displayed in (a–d) and (e), respectively. The symbols of ^∗^, ^∗∗^, and ^∗∗∗^ indicate the *P* values <0.05, 0.01, and 0.001, respectively.

As high immune cell infiltration was associated with high tumor burden and PLOD3 was associated with tumor genome instability, we then investigated the association of PLOD3 expression with immune cell infiltration. We first estimated the immune cell proportions in TCGA tumor samples using CIBERSORT (Materials and Methods). We found that the proportions of CD4 naïve and CD8 effector were negatively correlated with PLOD3 expression (Figures 5(a) and 5(c)), while the proportions of CD8 naïve and regulatory T cells (Tregs) were positively correlated with PLOD3 expression (Figures 5(b) and 5(d)). Consistently, these immune cells showed significantly different infiltrating levels between the high and low PLOD3 groups (Figures 5(a)–5(d)). Furthermore, we also estimated the immune scores for TCGA samples using the ESTIMATE method (Materials and Methods) and found that the immune score was negatively correlated with PLOD3 expression and higher in samples with low PLOD3 expression (Figure 5(e)). These results indicated that high PLOD3 expression was associated with reduced immune cell infiltrating levels in COAD.

### 3.6. PLOD3 Is Correlated with Immune Regulators and Tumor-Promoting Genes

**Figure 6:**
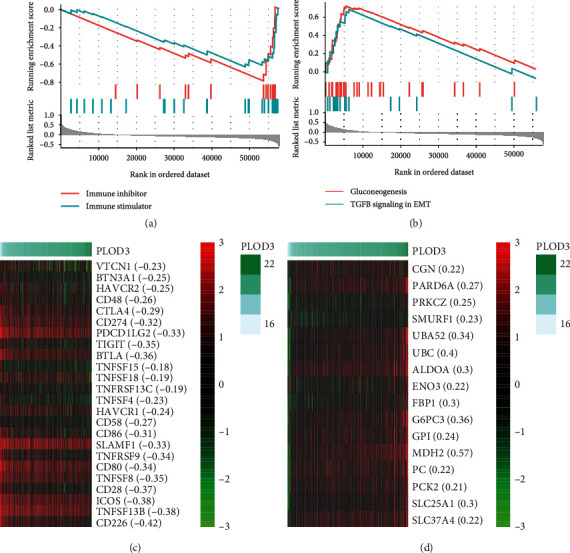
The association of PLOD3 expression with immune regulators and tumor-promoting pathways. The positively and negatively correlated genes with PLOD3 are enriched in immune inhibitors/stimulators and genes involved in tumor-promoting pathways including gluconeogenesis and TGF-beta signaling in epithelial-mesenchymal transition (EMT).

As PLOD3 was associated with immune cell infiltration, we then tested association of PLOD3 expression with immune regulators and investigated the downstream pathways regulated by PLOD3 using TCGA mRNA expression data. The correlation analysis was conducted between PLOD3 and the remaining genes, and the genes were preranked by Spearman's correlation with a decreasing order. The genes encoding immune stimulators and inhibitors and those involved in signaling pathways were collected from previous studies [20]. To test whether the genes involved in the pathways were significantly clustered within the positively or negatively correlated genes, we conducted gene set enrichment analysis on the preranked genes. Specifically, we found that both immune stimulators and inhibitors were enriched in genes negatively correlated with PLOD3 (Figure 6(a), FDR < 0.05). Moreover, the genes involved in two tumor-promoting pathways, including gluconeogenesis and TGF-beta signaling in epithelial-mesenchymal transition (EMT), were significantly enriched in those positively correlated with PLOD3 (Table 1 and Figure 6(b), FDR < 0.05). Consistently, the key genes involved in immune stimulation and inhibition showed reverse expression patterns with PLOD3 (Figure 6(c)), while those involved in gluconeogenesis and TGF-beta signaling in EMT, such as MDH2, G6PC3, ALDOA, TGFB1, and SMURF1, had similar expression patterns with PLOD3 (Figure 6(d) and Table 1). These results indicated that PLOD3 is correlated with immune regulators and tumor-promoting genes.

## 4. Discussion

Procollagen-lysine, 2-oxoglutarate 5-dioxygenases (PLODs) are a family of enzymes that regulate the hydroxylation of lysine and stabilization of collagen [21]. However, the clinical and functional roles of PLOD3 in COAD have not been investigated. The analysis of mRNA and protein expression data revealed that both mRNA and protein of PLOD3 were highly upregulated in COAD. The correlation analysis revealed that the promoter DNA methylation of PLOD3 was negatively correlated with its mRNA expression (Figure 1(d), Spearman correlation = −0.48). Consistently, the dysregulation of PLOD3 by aberrant DNA methylation has been found in melanoma [22]. Moreover, we also confirmed the upregulation of PLOD3 in COAD tissues using qPCR and Western blot (Figure 2). Furthermore, we also investigated the prognostic value of PLOD3 in COAD. PLOD3 was increased in the advanced TNM stages in both TCGA and CPTAC cohorts (Figures 3(a) and 3(b), *P* value <0.01), and high expression of PLOD3 indicated relatively worse prognosis in COAD. The association of PLOD3 with poor prognosis has been found in ovarian cancer [23], gastric cancer [24], and glioma [25].

As genome instability is a hallmark of cancer [26], we then investigated the association between PLOD3 and genomic instability such as chromosomal instability (CIN) and microsatellite instability (MSI) in COAD. PLOD3 was expressed higher in COAD samples with high chromosomal instability (CIN-high) than those with low CIN (CIN-low) and higher in those with low MSI than high MSI (Figure 4), indicating that PLOD3 expression was associated with tumor genomic instability. The acquisition of genomic instability is a crucial feature in colon cancer development [18] and can promote inflammatory signaling [27]. We found that immune cells showed significantly different infiltrating levels between the high and low PLOD3 groups, and the immune score was negatively correlated with PLOD3 expression and higher in samples with low PLOD3 expression (Figure 5), suggesting that high PLOD3 expression was associated with reduced immune cell infiltrating levels in COAD [28, 29]. To further uncover the underlying mechanism of PLOD3 in PLOD3, we compared the COAD samples of high PLOD3 expression with those of low PLOD3 expression and found that high expression of PLOD3 was associated with reduced expression of immune regulators and enhanced activities of two tumor-promoting pathways, including gluconeogenesis and TGF-beta signaling in epithelial-mesenchymal transition (EMT). The gluconeogenesis plays a role in signaling, proliferation, and the cancer stem cell (CSC) tumor phenotype [24]. Notably, TGF-beta signaling in EMT is a key player in angiogenesis, tumor growth, and metastasis in colon cancer [30], suggesting that PLOD3 might regulate immune microenvironment of COAD via TGF-beta signaling. In accordance with that, knockdown of PLOD3 inhibited HIF-1*α* accumulation via the ERK signaling pathway under hypoxia, suggesting that PLOD3 had the potential to regulate the tumor microenvironment [25].

Although the present study uncovered the expression pattern and potential clinical and scientific significances of PLOD3 in COAD, it still has some limitations. For example, the upstream and downstream relationship between the PLOD3 expression and genomic instability or immune cell infiltration needs further investigation. Moreover, the downstream pathways of PLOD3 in COAD should be validated by *in vivo/vitro* experiments. However, the present study still highlights the importance of PLOD3 and provides the evidence about the functional role of PLOD3 in COAD.

## Figures and Tables

**Table 1:** The statistical significance of core genes involved in EMT and gluconeogenesis.

ID	NES	*P* value	*P*.adjust	*q* values	Core_enrichment
Gluconeogenesis	3.00	2.32*E*-10	2.40*E*-08	1.03*E*-08	MDH2, G6PC3, ALDOA, FBP1, SLC25A1, SLC25A10, GPI, PC, ENO3, SLC37A4, PCK2, TPI1, SLC25A11, GAPDH, ALDOB, GOT2, ALDOC, PCK1, SLC25A13
TGFB signaling in EMT	2.27	1.40*E*-04	1.03*E*-03	4.39*E*-04	UBC, UBA52, PARD6A, PRKCZ, SMURF1, CGN, ARHGEF18, UBB, F11R, RPS27A, TGFB1

## Data Availability

The data that support the findings of this study are available from the corresponding author upon reasonable request.
